# A randomized clinical trial of intranasal dexmedetomidine versus inhaled nitrous oxide for procedural sedation and analgesia in children

**DOI:** 10.1186/s13049-024-01292-0

**Published:** 2024-11-20

**Authors:** Anna Nikula, Malin Ryd Rinder, Stefan Lundeberg, Mitja Lääperi, Katri Sandholm, Maaret Castrén, Lisa Kurland

**Affiliations:** 1 Department of Emergency Medicine and Services, Helsinki University, University of Helsinki, P.O. Box, Helsinki, 00014 Finland; 2 Department of Emergency Care for Children Astrid Lindgren Children´s Hospital, Department of Women’s and Children’s Health Karolinska Institutet, Stockholm, 17176 Sweden; 3 Department of Pediatric Anesthesia and Intensive Care, Department of Physiology and Pharmacology, Astrid Lindgren Children’s Hospital, Karolinska University Hospital, Karolinska Institutet, Stockholm, 17176 Sweden; 4 Lääperi Statistical Consulting, Helsinki, Finland; 5 Department of Emergency Medicine and Services, Helsinki University and Helsinki University Hospital, University of Helsinki, P.O. Box 4, Helsinki, 00014 Finland; 6 Department of Medical Sciences, Örebro University, Örebro, SE-701 82 Sweden

**Keywords:** Intranasal, Dexmedetomidine, Esketamine, Procedure, Sedation, Analgesia, Children

## Abstract

**Background:**

Procedural sedation and analgesia is an important part of pediatric emergency care, safe and clinically useful alternatives for adequate management are necessary. The objective of this clinical trial was to evaluate the non-inferiority of intranasal dexmedetomidine to nitrous oxide with respect to analgesia for a painful procedure in children 3–15 years of age.

**Methods:**

This prospective, equally randomized, open-label, non-inferiority trial was conducted at a Pediatric Emergency Department. Previously healthy children 3–15 years of age, with an extremity fracture or luxation or a burn and requiring procedural sedation and analgesia were eligible. Patients were randomized to receive either intranasal dexmedetomidine or inhaled nitrous oxide. The primary outcome measure was highest pain level during the procedure, assessed with Face, Legs, Activity, Cry, Consolability scale (FLACC). Mann-Whitney U test (continuous variables) and Fisher’s test (categorical variables) were used for statistical analysis.

**Results:**

The highest FLACC was median 4 (IQR 3–6) with intranasal dexmedetomidine and median 4 (IQR 2–6) with nitrous oxide. The median of the difference between samples from each group for FLACC was 0 with 95%CI (0–1), thus intranasal dexmedetomidine was not inferior to nitrous oxide with respect to the level of pain during the procedure. The same method for procedural sedation and analgesia would be accepted by 52/74 (82.5%) children and 65/74 (91.5%) parents in the intranasal dexmedetomidine group respectively 59/74 (88.1%) versus 70/74 (94.6%) with nitrous oxide. No serious adverse events were reported.

**Conclusions:**

The results of this trial support that intranasal dexmedetomidine is not inferior to 50% nitrous oxide in providing analgesia for a painful procedure in children 3–15 years of age and can be considered as an alternative to 50% nitrous oxide for procedural sedation and analgesia.

**Trial registration:**

EudraCT 201,600,377,317, April 20, 2017. https://eudract.ema.europa.eu/.

**Supplementary Information:**

The online version contains supplementary material available at 10.1186/s13049-024-01292-0.

## Background

Adequate procedural sedation and analgesia (PSA) enables the management of a wide range of procedures in the Emergency Department (ED). Fracture reduction is one of the most common procedures in pediatric emergency care and especially forearm and finger fractures can be managed successfully with closed reduction in the ED if adequate analgesia and sedation are ensured [1]. Fracture reduction in the ED is both cost-effective [2] and convenient for children and families.

Different drugs and administration routes are used for PSA. Intravenous administration is common [3, 4], but venous cannulation can cause distress [5] and other administration routes are therefore of interest. Drug delivery by inhalation is an alternative. Rapid onset and recovery along with a minimal effect on the cardiorespiratory system [6–10] make inhaled nitrous oxide (N_2_O) an ideal agent for PSA [11] and it is frequently used in pediatric EDs [4, 12]. Furthermore, it is routine in many EDs in Scandinavia, especially Denmark and Sweden [13]. However, the commonly used 50%N_2_O: 50%oxygen (50N_2_O) [4] does not provide adequate analgesia for painful procedures as a single agent, but in combination with hematoma block good analgesic effect has been shown [14–16]. In addition, the use of N_2_O is limited by medical conditions e.g., ear infection, respiratory tract infection. Poor tolerability of the mask additionally limits its use. Consequently, there is a need for other safe and clinically useful alternative agents for PSA.

Another non-invasive route is intranasal (IN) administration, which is practical in pediatric care. Several drugs, including dexmedetomidine, used intranasally cause minimal discomfort in contrast to i.e. midazolam which can cause sharp reactions. IN dexmedetomidine (DEX), an alpha-2 adrenergic receptor agonist, has sedative, anxiolytic and analgesic effects [17]. Furthermore, it is an attractive drug for PSA as it rarely has clinically significant cardiorespiratory effects [18–24]. Good analgesic and sedative effect during painful procedures has been reported with venous cannulation [19], laceration repair [25] and dental treatment [22], but further evidence is needed for more painful procedures, e.g. fracture reduction.

The objective of this clinical trial was to evaluate the non-inferiority of IN DEX to 50N_2_O with respect to analgesia for a painful procedure among children 3–15 years of age. Our primary outcome measure was pain, measured as the highest assessed pain level during the procedure.

## Methods

### Trial design

This non-inferiority, prospective, equally randomized (1:1), open-label, parallel-group clinical trial was conducted at a large urban pediatric ED. This trial was approved by the Regional Ethical Review Board and registered with the European Clinical Trial Registry. An independent regulatory unit monitored the trial. The trial protocol is presented as Supplementary material file 1.

The CONSORT guidelines [26] for reporting were followed.

## Participants

### Eligibility criteria for participants

Swedish speaking, previously healthy children 3 to 15 years of age with an extremity fracture or luxation needing reduction or a burn covering less than 4% of the body surface area and requiring PSA were eligible for this trial. ED physician (predominantly physicians in training in pediatrics, emergency medicine or general medicine) assessed the injury, and the need for PSA. Patient enrollment was possible when a trial physician was available. Exclusion criteria were: American Society of Anesthesiologist physical status classification (ASA) ≥ III [27], ongoing respiratory tract infection, ear infection, sinusitis, pertussis within 6 months, breathing difficulty, ongoing vomiting, reduced level of consciousness, mental health issues, hypersensitivity for dexmedetomidine or N_2_O, heart block (grade 2 or 3) unless paced (ECG was evaluated), uncontrolled hypotension, acute cerebrovascular conditions. Both written and verbal trial information was presented in Swedish to the parents and to the children at their level of understanding. Signed informed consent was given by the parents prior to inclusion.

### Setting and location

This clinical trial was conducted in the pediatric ED at Astrid Lindgren Children’s hospital, Karolinska University Hospital in Stockholm, Sweden, where medical care is provided annually to approximately 50 000 children and adolescents with all levels of injuries and illness.

### Interventions

The plan for PSA was finalized by the ED team caring for the patient after enrollment and randomization and information of the trial drug. The procedures included in the trial were reduction of a fracture, luxation or wound debridement and dressing for burns. Procedures were performed by ED physicians.

All patients received oral paracetamol (40 mg/kg) no later than 1–1.5 h before the procedure, dose administered prior to ED arrival was taken into consideration when ordering paracetamol. N_2_O was administered with a facial mask held by an ED nurse certified for N_2_O sedation. N_2_O was titrated to a concentration of 50%N_2_O: 50%O_2_ within 2–3 min. 100% O_2_ was given for 2–3 min after discontinuation of N_2_O. Dexmedetomidine 100 mcg/mL was used without dilution and administered with a nasal atomizer. IN DEX dose 2.0 mcg/kg was used following the local and national guidelines [28]. The dose was divided equally between both nostrils for children weighing > 15 kg in order not to exceed the recommended volume per nostril (0.3mL/nostril) [29].

Buffered lidocaine (10mL 1% lidocaine + 2mL NaHCO_3_ 0.6 M) was used for local anesthesia. For fractures, a hematoma block was performed and for luxations lidocaine was infiltrated into the joint 5 min before the procedure. Maximum dose of lidocaine without adrenaline was 5 mg/kg and with adrenaline 7 mg/kg.

The procedure was started when adequate sedative effect of the trial drug was reached. Adequate sedation level was defined as Ramsay 2 (= awake; co-operative, orientated and tranquil or babbling, laughing when sedated with N_2_O). If sufficient sedation was not reached within 30 min, the procedure was started if the child was co-operative. When the child was not co-operative the trial was terminated.

### Outcomes

The primary outcome measure was the highest level of pain during the procedure. Secondary outcomes were sedation depth, patient and parental satisfaction and assessment of pain during the procedure, and ED physician’s opinion of the feasibility of the procedure.

All assessments and observations as described below were performed by two experienced pediatricians (AN, KS). The data was entered into the Case Report Form, which was reviewed by the principal trial physician (AN) and the monitor from the independent regulatory unit.

Patients were monitored and observed continuously from the time of administration of the trial drug until recovery, i.e. Ramsay score 1. Pain and sedation depth as well as vital signs were actively assessed by the trial physician and reported every 5 min before and during the procedure and every 10 min after the procedure until recovery.

Vital signs, oxygen saturation (SpO_2_) and heart rate, were monitored continuously and recorded at the same timepoints as FLACC and Ramsay unless deviation from normal vital signs, as described in Pediatric Advanced Life Support [30], occurred. Adverse events were reported following the definitions and recommendations proposed by the Consensus Panel on Sedation of Pediatric Emergency Research Canada and the Pediatric Emergency Care Applied Research Network [31].

Level of pain was assessed using the Face, Legs, Activity, Cry, Consolability scale (FLACC) validated and recommended for procedural pain assessment [32–34]. Sedation depth was assessed with the Ramsay sedation scale [35], which is a widely used tool for observational sedation assessment and has previously been used in studies assessing sedation with IN DEX [20, 21]. Ramsay score 1 = awake, 2 = awake; co-operative, orientated and tranquil or babbling, laughing when sedated with N_2_O, 3 = awake; responds to commands only, 4 = asleep; reacts with a brisk response to a light glabellar tap or a loud auditory stimulus, 5 = asleep; reacts with a sluggish response to a light glabellar tap or a loud auditory stimulus, 6 = asleep; does not respond to pain.

Patient and parental satisfaction was evaluated with a questionnaire which was distributed after the procedure. The patient questionnaire was composed of two questions: (1) how much pain on a scale of 0–10 did you have during the procedure. A revised Faces Pain Scale (FPS-R) [36] was shown to the patient. (2) If same treatment is needed in the future, would the same method for sedation be acceptable? Yes / no. If the child was not old enough to understand written questions, the trial physician presented the questions. Parent/parents, who were present from the administration of the trial drug until recovery received a questionnaire with three questions: (1) in your opinion; how much pain on a scale of 0–10 did your child have during the procedure (the FPS-R was shown to the parents), (2) What is your opinion of the general management of the analgesia and sedation and the procedure on a scale of 1–5 (1 = not at all satisfied, 5 = very satisfied), (3) if your child needed the same treatment in the future, would the same method for sedation be acceptable? Yes / no. The ED physicians graded the feasibility of performing the procedure on a scale of 1–5 (1 = very easy, 5 = very difficult).

### Randomization

Patients were randomized equally (1:1) to two groups: (1) IN DEX 2.0 mcg/kg, (2) inhaled 50N_2_O. We used block randomization (blocks of 10 subjects (5 from both arms), except one block with 6 subjects). A randomization list, after a random draw, was created by a physician not participating in the current trial. Opaque envelopes were filled with information (trial drug and IN DEX dose table) and numbered according to the list. Envelopes were used in number order. The blocks were used in order which was not known by the physician performing the assessment of the patient. The randomization list was sealed in an envelope and securely stored during trial period and the seal was verified to be intact at the time of trial closure.

### Statistical methods

An a priori power analysis was conducted to test the null hypothesis that IN DEX is non-inferior to 50N_2_O with respect to pain assessed with FLACC. A difference of 1 point on the FLACC scale was considered as the smallest clinically relevant difference. The power calculation was done with an assumption of a 2.50 within-group standard deviation which resulted in needing 78 patients per group to obtain 0.80 power to reject the null hypothesis with alpha set to 0.05.

Continuous variables are presented using medians and interquartile ranges (IQRs) and tested with Mann-Whitney U test and tested correlations using the Spearman method. Categorical variables are presented using counts and percentages and tested using Fisher’s test. As the outcome was non-normally distributed the non-inferiority was investigated using median of the differences between the samples from each group and their 95% confidence intervals. We considered p-values below 0.05 significant. All analyses were done using R version 4.3.2 [37].

## Results

### Participants

**Fig. 1:**
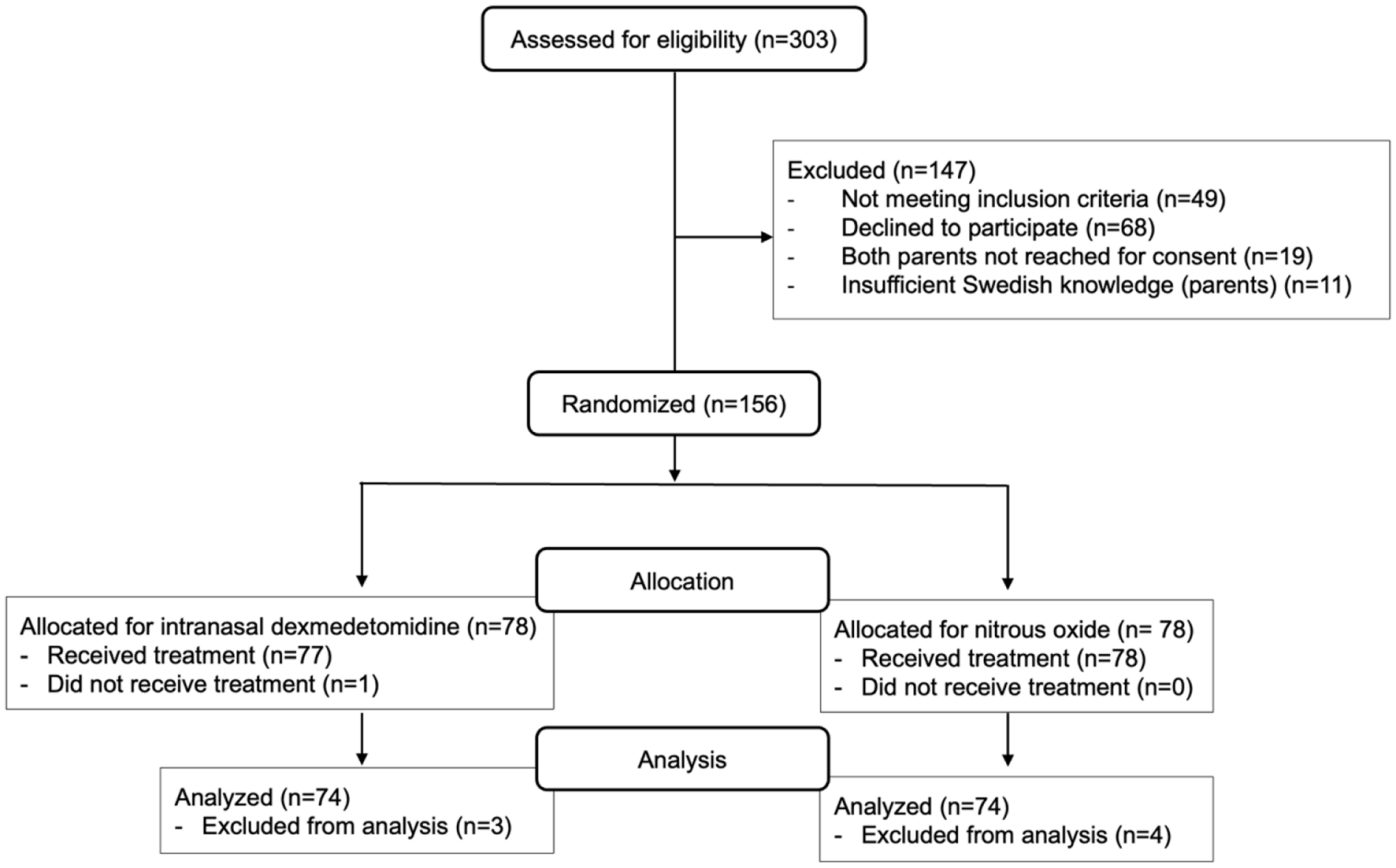
Participant flow chart

Patient enrollment was performed between August 2017 and September 2020. 303 patients were assessed for eligibility and 156 patients were randomized, see trial flow chart (Fig. 1). Eight patients did not follow protocol. One who was planned for fracture reduction in the ED and had already been included in the trial was after consultation with senior orthopedic surgeon not treated in the ED but underwent surgery. Three patients in group IN DEX and one in group 50N_2_O did not reach Ramsay level 2 and were not co-operative. Additional three patients did not undergo the procedure with N_2_O due to: one patient did not tolerate the mask, one had tinnitus and one continued vomiting despite antiemetic drug. In total, 148 patients (74 in each group) received adequate analgesia and sedation to complete the procedure in accordance with the trial protocol. The groups were similar regarding baseline demographics (Table 1). The most common injury type was forearm fracture, no patients with burns were enrolled in the trial.

**Table 1:** Baseline demographics among patients that followed protocol

	All	Intranasal dexmedetomidine	Nitrous Oxide
**Number of patients**	148	74	74
**Age (years)** median (IQR)	8.9 (6.8–12.0)	9.3 (6.8–12.0)	8.7 (6.7–12)
**Weight (kg)** median (IQR)	32.0 (24.0–45.2)	32.3 (24.0–45.5)	32.0 (24.9–45.0)
**Sex**			
Male (%)	84 (56.7)	44 (59.5)	40 (54.1)
Female (%)	64 (43.2)	30 (40.5)	34 (45.9)
**Type of Injury (%)**			
Fracture	145	73	72
Radius and ulna	68 (45.9)	36 (48.6)	32 (43.2)
Isolated radius or ulna	69 (46.6)	32 (43.2)	37 (50.0)
Finger/toe	8 (5.4)	5 (6.8)	3 (4.1)
Luxation elbow	3 (2.0)	1 (1.4)	2 (2.7)
Burn	0	0	0

### Main results

**Fig. 2:**
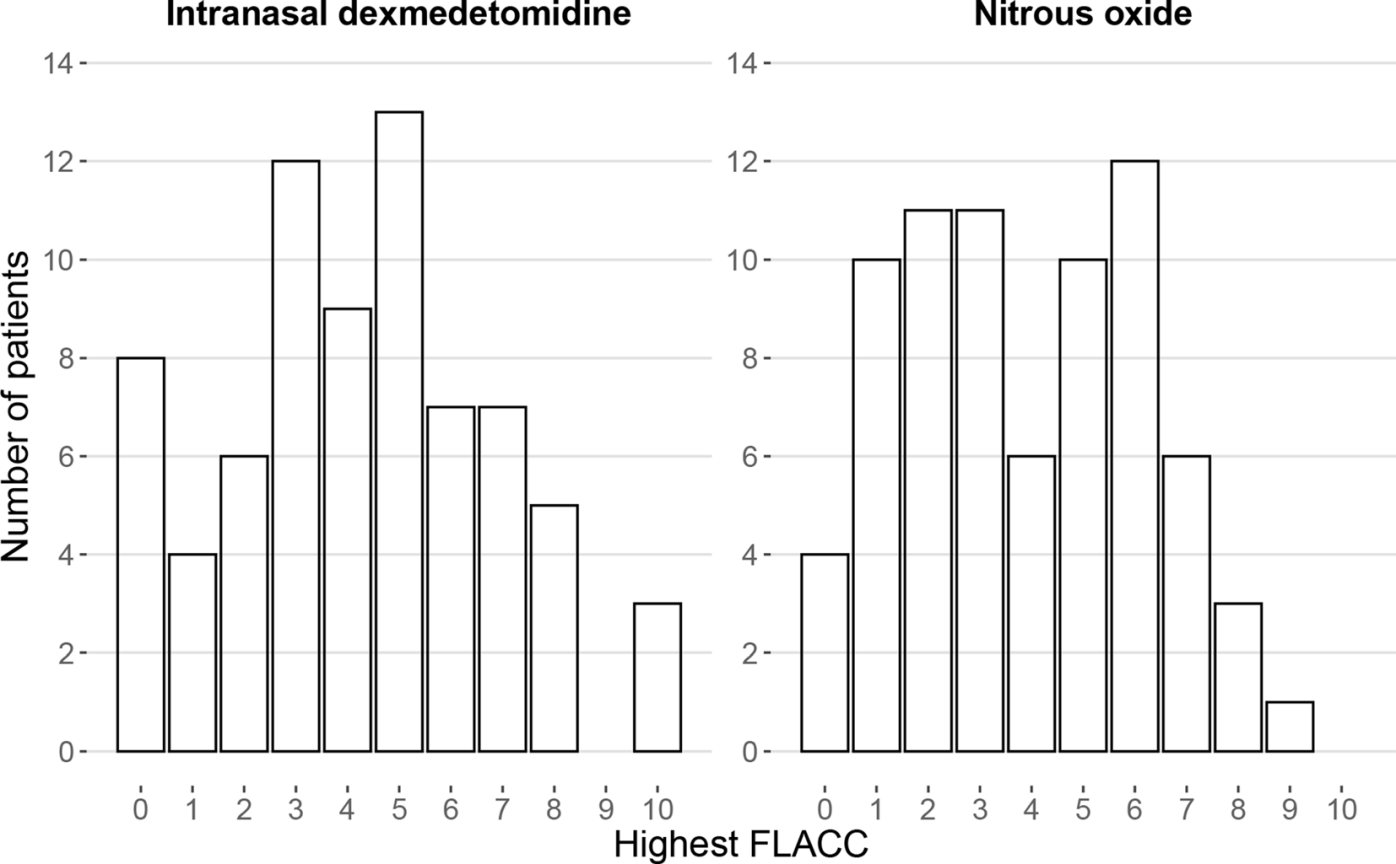
Highest pain level during the procedure. Pain level was assessed with Face, Legs, Activity, Cry, Consolability scale (FLACC). This diagram shows the distribution of FLACC scores per drug. The median (IQR) of highest FLACC with patients receiving intranasal dexmedetomidine was 4 (3-6) and nitrous oxide 4 (2-6), p-value 0.41. The median of the difference between samples from each group for FLACC was 0 with 95%CI (0–1) which is within the predefined limit for non-inferiority

All patients had FLACC 0 before administration of the trial drug. The highest FLACC during the procedure was median (IQR) 4 (3-6) in the IN DEX treated group and 4 (2-6) in the group treated with N_2_O. The distribution of highest FLACC scores per group are shown in Fig. 2. The median of the difference between samples from each group for FLACC was 0 with 95%CI (0–1) which is within the predefined limit for non-inferiority. Hence, IN DEX was not inferior to N_2_O with regard to FLACC. The pairwise comparison using Mann-Whitney U test also reflects this relationship, as it has a p-value of 0.41, i.e. no significant difference was observed. Table 2 shows the comparisons between groups for the highest pain and sedation level during the procedure.

**Table 2:** Comparisons of pain and sedation levels between intranasal dexmedetomidine and 50% nitrous oxide. The highest level of pain (FLACC) and deepest level of sedation (Ramsay) during the procedure are presented. Continuous variables (FLACC) are presented using medians and IQRs. Categorical variables (Ramsay) are presented using counts and percentages

	Levels	Intranasal dexmedetomidine (*n* = 74)	50% nitrous oxide (*n* = 74)	*p*-value
**Highest FLACC** Median (IQR)		4 (3–6)	4 (2–6)	0.41
**Highest Ramsay** n (%)	1	3 (4.1%)	0	< 0.001
	2	54 (73.0%)	74 (100%)	
	3	14 (18.9%)	0	
	4	3 (4.1%)	0	
	5	0	0	
	6	0	0	

*Abbreviations* FLACC = Face, Legs, Activity, Cry, Consolability scale

**Fig. 3:**
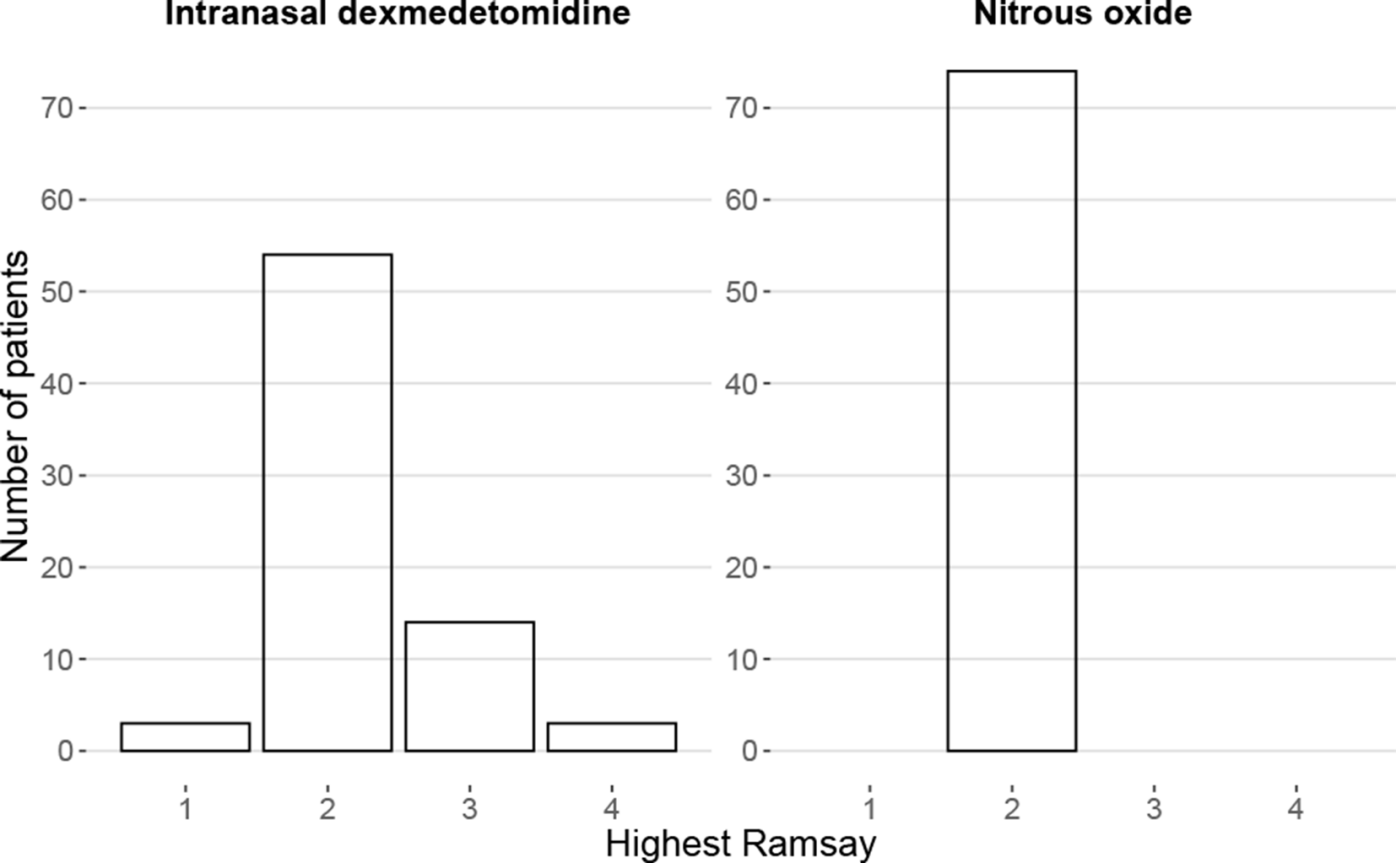
Highest sedation scores during the procedure. Sedation level was assessed with Ramsay sedation scale. This diagram shows the distribution of Ramsay scores per drug. The groups differ significantly, p-value <0.001.

Sedation level Ramsay ≥ 2 was reached in 71/74 (95.9%) patients receiving IN DEX. Three patients (4.1%) had Ramsay 1, they were co-operative, and the procedure could be completed. 54 (73.0%) patients had Ramsay 2, 14 (18.9%) Ramsay 3 and three (4.1%) patients Ramsay 4. Ramsay 2 was reached in 74/74 (100%) patients treated with N_2_O (Table 2). The groups differ significantly due to patients reaching higher Ramsay score with IN DEX, Fisher’s test showed a p-value < 0.001. Distribution of sedation levels is shown in Fig. 3.

The median (IQR) for FPS-R as graded by the patients was 5.5 (2–8) in group IN DEX and 4 (2–6) in group 50N_2_O, p-value 0.048. Parents’ assessment of their child’s pain median (IQR) FPS-R was 6 (2–8) in group IN DEX and 3 (1–7) in group 50N_2_O, p-value 0.007. In group IN DEX 52 (82.5%) patients and 65 (91.5%) parents would accept the same method for PSA in the future in comparison to 59 (88.1%) patients and 70 (94.6%) parents in group 50N_2_O. No significant difference was shown between the groups in patient’s (p-value 0.46) or parents’ acceptance (p-value 0.53). In total 133/148 parents were satisfied or very satisfied with the general management of the analgesia and sedation and the procedure: 64 (87.7%) parents of children receiving IN DEX and 69 (94.5%) parents in group 50N_2_O, p-value 0.094. The ED physicians graded the feasibility of the procedure easy or very easy in 58 (78.4%) cases in the group treated with IN DEX and in 63 (85.1%) treated with 50N_2_O. In 2 (2.7%) cases with patients receiving IN DEX the ED physician graded the procedure as very difficult and in no cases with patients in group 50N_2_O. Comparisons between the groups are presented in Table 3.

**Table 3:** Comparisons between intranasal dexmedetomidine and nitrous oxide. Patient and parent questionnaires and ED physician’s opinion are presented in this table. Continuous variables are presented using medians and IQRs. Categorical variables are presented using counts and percentages

	Intranasal dexmedetomidine		50% Nitrous oxide		**p-value**
		Missing (n)		Missing (n)	
**FPS-R by patient**	5.5 (2–8)	8	4 (2–6)	15	0.048
**FPS-R by parent**	6 (2–8)	1	3 (1–7)	0	0.007
**Same method acceptable in future /patient**	52 (82.5%)	12	59 (88.1%)	7	0.46
**Same method acceptable in future /parent**	65 (91.5%)	3	70 (94.6%)	0	0.53
**Parental satisfaction with the general management**		1		1	
*1 = Not at all satisfied*	0		0		0.094
*2 = Not satisfied*	3 (4.1%)		0		
*3 = Neutral*	6 (8.2%)		4 (5.5%)		
*4 = Satisfied*	19 (26.0%)		12 (16.4%)		
*5 = Very satisfied*	45 (61.6%)		57 (78.1%)		
**Feasibility by ED physician**		0		0	
*1 = Very easy*	42 (56.8%)		44 (59.5%)		0.66
*2 = Easy*	16 (21.6%)		19 (25.7%)		
*3 = Neutral*	10 (13.5%)		9 (12.2%)		
*4 = Difficult*	4 (5.4%)		2 (2.7%)		
*5 = Very difficult*	2 (2.7%)		0		

*Abbreviations* FPS-R = Faces Pain Scale revised, ED = Emergency Department

Sedation level was measured every 5 to 10 min according to trial protocol. Time to start of the procedure, i.e. reaching Ramsay 2 was median (IQR) 15.0 (15.0–20.0) minutes for IN DEX and 5.0 (5.0–10.0) minutes for 50N_2_O. Time to recovery was median (IQR) 30.0 (10.0–40.0) minutes for IN DEX and 0.0 (0.0–0.0) minutes for 50N_2_O.

### Adverse events

No serious adverse events were reported. Three patients receiving IN DEX had briefly SpO_2_ between 90 and 95%, no interventions were required. Seven patients in the group IN DEX had bradycardia, none required any intervention. Six patients (8.1%) sedated with 50N_2_O were reported having nausea and vomiting, none among those treated with IN DEX. Antiemetic drug was administered to all patients who vomited and N_2_O was restarted after a pause. None of these patients had complications due to vomiting after the pause and N_2_O was restarted. Four (5.4%) patients in group IN DEX and one (1.4%) in group N_2_O experienced perceptional disturbances (e.g. hallucinations, dreamlike state), no interventions were required. Adverse events prevented two patients from continuing N_2_O sedation; one due to repeated vomiting despite of antiemetic administration and one patient suffered from amplified voices and tinnitus. These symptoms vanished quickly after discontinuation of N_2_O. (Table 4.)

**Table 4:** Adverse events. All adverse events reported during the trial, including those preventing from continuing sedation (two patients receiving nitrous oxide) presented in separate column

	Intranasal dexmedetomidine (*n* = 74)	50% Nitrous oxide (*n* = 74)	Adverse events preventing patients from continuing sedation with trial drug
**Cardiorespiratory**	**10 (13.5%)**	**0**	
*Oxygen saturation < 95%*	3 (4.1%)	0	
*Oxygen saturation < 95% and requiring intervention*	0	0	
Bradycardia*	7 (9.5%)	0	
Bradycardia* *and requiring intervention*	0	0	
**Neurology**	**4 (5.4%)**	**1 (1.4%)**	
*Hallucinations/dreamlike state*	4 (5.4%)	1 (1.4%)	
*Amplified voices / tinnitus*	0	0	1 N_2_O
**Gastrointestinal**	**0**	**6 (8.1%)**	
*Nausea and vomiting*	0	6 (8.1%)	1 N_2_O

* Bradycardia as described in Pediatric Advanced Life Support [30]

*Abbreviations* N_2_O = nitrous oxide

## Discussion

The results of this clinical trial show that intranasal dexmedetomidine is not inferior to 50N_2_O assessed with FLACC and adequate sedation can be provided during a painful procedure for children 3–15 years of age. Most of the patients and parents would accept intranasal dexmedetomidine for procedural sedation and analgesia in the future and high parental satisfaction with intranasal dexmedetomidine for sedation and analgesia was reported. These results support that intranasal dexmedetomidine can be used as an alternative to 50N_2_O and may facilitate an individualized management of procedural sedation and analgesia.

The current trial demonstrated the non-inferiority of IN DEX to 50N_2_O during a painful procedure with respect to pain assessed with FLACC. To our knowledge, there are no previous trials comparing these two drugs. The analgesic effect of IN DEX in children has previously been studied during venous cannulation [19], laceration repair [25] and dental treatment [22]. FLACC levels reported during venous cannulation and laceration repair were lower than in this current trial, which was to be expected as these procedures are generally considered less painful than fracture reduction. Patients receiving IN DEX for dental treatment showed similar pain levels as patients during fracture reduction in this current trial. The results show that IN DEX in combination with local anesthesia and oral paracetamol provide adequate analgesia during fracture or luxation reduction.

Almost all patients reached an adequate level of sedation with 2.0 mcg/kg IN DEX. Similar results were reported with the use of 2.0 mcg/kg IN DEX for dental treatment [22]. However, Poonai et al. found only every fifth child adequately sedated with 2.0 mcg/kg for laceration repair and they concluded that IN DEX 3.0 or 4.0 mcg/kg would be optimal dose to use for laceration repair [38]. However, different assessment tools for sedation depth were used in the study by Poonai et al. and deeper sedation was considered necessary as compared to both the current trial and in the trial for dental treatment [22], hence the results are not directly comparable. Previously the sedative effect of IN DEX has been widely demonstrated for non-painful procedures in children [20, 21, 39, 40]. The results of the current trial provide additional support for the adequate sedative effect of IN DEX also during painful procedures in children.

In the current trial both patients and parents graded higher pain levels with IN DEX than 50N_2_O which differs from the pain levels assessed by the trial physicians. A similar discrepancy between pain scoring by child, parent and health care professionals has been reported [41, 42]. A few explanations for this have been discussed; e.g., health care professionals have seen patients in different levels of pain and relate their assessment to these experiences, patients relate their pain to their own previous experiences which may vary greatly [41, 42]. However, pain is a personal experience [43] and therefore the results in the current study should not be dismissed. But to ensure uniform pain assessment we chose to use FLACC by trial physician. Our choice was further impacted by the understanding that sedated patients cannot report their pain and pain assessments cannot be done retrospectively due to the uncertainty of how pain is remembered afterwards [44].

Most of the patients and parents, in both groups, would accept the same method for PSA in the future. In addition, most parents were satisfied or very satisfied with the management of the analgesia and sedation and the procedure in both groups. Similar high parental satisfaction with PSA in general, with various drugs, has been reported [45]. Furthermore, provider satisfaction is important when assessing the adequacy of PSA. In our current trial ED physicians graded the feasibility of performing the procedure as easy or very easy in the majority of cases, and to a similar extent in both groups. Comparable high provider satisfaction has been reported for IN DEX in PSA during different types of procedures [25, 46]. These findings support the use of IN DEX for PSA during painful procedures in children.

No serious adverse events or significant cardiorespiratory effects were reported in this current trial. Nausea and vomiting were the most common adverse events with 50N_2_O, which is in-line with previous reports [7, 15]. Perceptual disturbances, described as a dreamlike state, were the most reported adverse events for treatment with IN DEX. Interestingly, there are to our knowledge no previous reports describing this phenomenon, and therefore we speculate that this phenomenon may be considered to be expected when using IN DEX. Adverse events with 50N_2_O and IN DEX were self-limiting and required no interventions with the exception of antiemetic treatment for vomiting.

IN DEX and N_2_O induce different types of sedation, with different contraindications, time to sedation onset and recovery, and require different settings, e.g. staffing and equipment. These differences are the basis for the possibility to choose the most suitable drug for each patient and situation. Administration of N_2_O requires co-operation and acceptance of the inhalation mask, therefore IN DEX can be more suitable for some children, especially the younger children. N_2_O provides faster onset and recovery than IN DEX and can therefore shorten the ED stay, which might be beneficial in an over-crowded ED. On the other hand, administration of N_2_O requires a dedicated and certified staff member throughout the procedure which maybe a limiting factor. Choosing the most suitable drug, the child’s perspective should be taken into consideration. Especially older children may have preferences on the type of sedation they wish. After careful explanation of possibilities, a joint decision could be reached.

### Limitations

The level of pain experienced by the children may have been influenced by the level of experience of the ED physician performing hematoma block and reduction as the procedures were performed by the ED physician on duty and responsible for the individual patient. However, we believe that the design may be viewed as representative of many EDs as the physicians on duty have varying levels of expertise, hence supporting the generalizability of the results.

The type of sedation produced by N_2_O and IN DEX are different, which challenges the assessment of sedation depth. N_2_O is a dissociative sedative and while it can make children tranquil, more often they become relaxed and start laughing and babbling. Conversely, IN DEX renders a natural sleep-like sedation [47]. To achieve a reliable and reproducible assessment of the sedation level, we added babbling and laughing to the original description of Ramsay level 2.

Trial physicians were not blinded to the treatment the patient received which was a limitation and could potentially lead to bias with respect to the assessment of pain and sedation. Blinding was not possible due to the different method of delivery and the type of sedation produced by the drugs. Alternatives of blinding the assessors, e.g. videotaping the procedure, wound not have resolved the lack of blinding as the assessment involves an evaluation of facial expression, especially FLACC, and therefore the delivery method (N_2_O is delivered by mask) would have been visible to the assessor. Even if using a mask to disguise the delivery of DEX the drug administered would have been evident for the assessor as DEX and N_2_O produce different types of sedation, time of onset and recovery. Furthermore, we acknowledge that mask for N_2_O delivery may have affected the perception of vocalizations and changes in facial expression, which could have altered the assessment of pain scores with FLACC by trial physician. Two independent assessors could have increased the reliability of the assessments, but this was not possible due to a general shortage of experienced pediatric emergency physicians.

The level of sedation was assessed every five minutes before and every ten minutes after the procedure, therefore the time to sedation and recovery are given at these intervals and the results should therefore only be used as an estimate for the time required to sedation onset and recovery. However, these times are within the same range as previously reported [18, 48].

### Generalizability

These results cannot be generalized to an age group other than that of children corresponding to those included in this current trial. In particular, the results should not be applied to PSA for children under three years of age or with underlying medical conditions. Fracture and luxation reduction are among the most painful procedures in pediatric emergency care, so we therefore believe that the results can be applied to PSA during other procedures, e.g. laceration repair, wound debridement.

## Conclusion

In conclusion, the results of this clinical trial support that IN DEX is not inferior to 50% nitrous oxide in providing analgesia for a painful procedure among children 3–15 years of age. Furthermore, IN DEX can be considered as an alternative to 50N_2_O for procedural sedation and analgesia in the emergency department.

## Electronic supplementary material

Below is the link to the electronic supplementary material.

Supplementary Material 1

## Data Availability

The datasets used and analyzed during the current study are available from the corresponding author on reasonable request.

## Abbreviations

ASA: American Society of Anesthesiologist physical status classification
DEX: Dexmedetomidine
ED: Emergency Department
FLACC: Face, Legs, Activity, Cry, Consolability scale
FPS-R: revised Faces Pain Scale
HR: Heart rate
IN: Intranasal
IQR: Interquartile range
N_2_O: Nitrous oxide
50N_2_O: 50% nitrous oxide:50% oxygen
PSA: Procedural sedation and analgesia
SpO_2_: Oxygen saturation
