# Supplementary material for: A randomized clinical trial of intranasal dexmedetomidine versus inhaled nitrous oxide for procedural sedation and analgesia in children
Source: Scand J Trauma Resusc Emerg Med. 2024 Nov 20;32:117. doi: 10.1186/s13049-024-01292-0 (PMC11577660; doi:10.1186/s13049-024-01292-0)
Supplement: Supplementary file 1 — Supplementary Material 1 [file 13049_2024_1292_MOESM1_ESM.pdf]

Clinical Trial Protocol

A prospective randomized open label study
Intranasal dexmedetomidine versus inhaled nitrous oxide for children age 3 – 15 years for procedural sedation and analgesia in pediatric emergency department.

Eudra CT nr 2016-003773-17A dex_version1 version 4

Resubmission A

2017-06-21

ABBREVIATIONS

ALB	Astrid Lindgren Children's hospital
ASA	American Society of Anesthesiologist physical status classification system
ED	Emergency Department
FLACC	Facies, Legs, Activity, Cry, Consolability -pain scale
IN	Intranasal
IV	Intravenous
N_2O	Nitrous Oxide
PALS	Pediatric Advanced Life Support (guidelines from American Heart Association)
PSA	Procedural Sedation and Analgesia
SpO_2	Oxygen saturation

INDEX

Synopsis 5

6.1 General Information 7

6.1.2 sponsor
6.1.3 person authorized to sign the protocol
6.1.4 the sponsor's medical expert for the trial
6.1.5 investigator responsible for conducting the trial
6.1.6 physician responsible for trial-site related medical decisions

Informed Consent of Trial Subjects 9

Records and Reports 9

6.2 Background Information 10

6.2.1 Name and description of the investigational product(s).
6.2.2 A summary of findings from studies that are relevant to the trial
6.2.3 Summary of the known and potential risks and benefits
6.2.4 Route of administration, dosage, dosage regimen, and treatment period.
6.2.5 A statement that the trial will be conducted…
6.2.6 Description of the population to be studied.
6.2.7 References to literature and data

6.3 Trial Objectives and Purpose 13

6.4 Trial Design 14

6.4.1 A specific statement of the primary endpoints and the secondary endpoints
6.4.2 A description of the type/design of trial
6.4.3 A description of the measures taken to minimize/avoid bias
(a) Randomization.
6.4.4 A description of the dosage and dosage regimen of the investigational product
6.4.5 The expected duration of subject participation
6.4.6 A description of the "stopping rules" or "discontinuation criteria"
6.4.7 Accountability procedures for the investigational produc and comparator
6.4.8 Maintenance of trial treatment randomization codes and procedures for breaking codes.
6.4.9 The identification of any data to be recorded directly on the CRFs

6.5 Selection and Withdrawal of Subjects 18

6.5.1 Subject inclusion criteria.
6.5.2 Subject exclusion criteria.
6.5.3 Subject withdrawal criteria and procedures specifying

6.6 Treatment of Subjects 19

6.6.1 The treatment(s) to be administered,
6.6.2 Medication permitted (including rescue medication) and not permitted
6.6.3 Procedures for monitoring subject compliance.

6.7 Assessment of Efficacy 20
6.7.1 Specification of the efficacy parameters.
6.7.2 Methods and timing for assessing, recording, and analysing of efficacy parameters.

6.8 Assessment of Safety 21
6.8.1 Specification of safety parameters.
6.8.2 The methods and timing for assessing, recording, and analysing safety parameters.
6.8.3 Procedures for eliciting reports of and for recording and reporting adverse event
6.8.4 The type and duration of the follow-up of subjects after adverse events.

6.9 Statistics 23
6.9.1 A description of the statistical methods
6.9.2 The number of subjects planned to be enrolled.
6.9.3 The level of significance to be used.
6.9.4 Criteria for the termination of the trial.
6.9.5 Procedure for accounting for missing, unused, and spurious data.
6.9.6 Procedures for reporting any deviation(s) from the original statistical plan
6.9.7 The selection of subjects to be included in the

6.10 Direct Access to Source Data/Documents 25

6.11 Quality Control and Quality Assurance 25

6.12 Ethics Description of ethical considerations relating to the trial 25

6.13 Data Handling and Record Keeping 25

6.14 Financing and Insurance 26

6.15 Publication Policy 26

6.16 Supplements 26

Signatures 27

Synopsis

Eudra CT nr 2016-003773-17A dex_version1, version 3

A prospective randomized study
Intranasal dexmedetomidine versus inhaled nitrous oxide for procedural sedation and analgesia in pediatric emergency department.

Injuries are common reasons for a visit to the pediatric emergency department (ED). In the US 24,7% of the pediatric ED visits were injury-related between 2004 and 2008, of which 76,9% were characterized as minor injuries (e.g. fractures, lacerations, burns, contusions, sprains).[1] In a Swedish analysis the rate of injury related visits to the ED was 110/1000 person years, of which approximately 60% required active treatment.[2]

Trauma often causes pain and anxiety and a visit to the hospital may cause further anxiety. The therapeutic procedures are usually frightening for the child and often also painful. Although pain can be treated with acetaminophen, cox-inhibitors and opioids as well as local anesthetics.[3] But the child's fear of further pain or the anxiety concerning the unfamiliar procedure cannot be sufficiently relieved with these analgelsics. The absence of good analgosedation (i.e. analgesia and sedation) can result in a very negative experience for both the child and caregivers and may also have impact on any procedures in the future.[4]

The possibility of performing the necessary procedures in the ED would reduce the need for hospital admissions and as a result reduce overall costs and the use of healthcare resources. But even more important, it is usually more convenient for the children and families to receive the required treatment in the ED when possible.

Procedural pain and anxiety has been treated successfully for several years in many pediatric EDs[5,6,7] following the goals and guidelines set for procedural sedation and analgesia (PSA)[8,9]. Commonly intravenous (IV) drugs (e.g. ketamine, propofol, etomidate) are used, ketamine[10] being the most widely reported agent. The safety and adverse effects of ketamine are well reported.[11,12] The use of intravenous sedative agents require a sound knowledge of the pharmacological effects of these drugs. The physician also need to be able to manage a compromised airway as well as rescue patients from inadvertent deep sedation. In Sweden pediatric EDs are not always staffed with physicians (i.e. pediatric emergency physicians/emergency physicians) with the necessary skill set, as in those countries where PSA is successfully used, hence we need to identify other medications to reach the same goals that are defined for PSA[8] i.e. adequate and safe analgosedation.

At the ED in Astrid Lindgren Children's hospital (ALB) nitrous oxide (N_2O) is routinely used for PSA for children between 3 and 15 years of age. Inhaled N_2O[13] is an ideal agent for procedural sedation[14]. It is safe and easy to administer. N_2O has rapid onset and recovery. Its effect on the cardiovascular and respiratory systems are minimal and the safety has been demonstrated in several studies.[15,16] The most commonly used concentration of 50% N_2O/ 50% O_2 mixture renders a mild to moderate sedation and mild analgesic effect. N_2O as a single agent does not provide adequate analgesia for more painful procedures.[17] However in combination with local anesthesia (e.g. hematomablock with fracture reduction) N_2O has shown a good effect on distress and memory of pain caused by the procedure.[18] Use of N_2O is limited by certain medical conditions (e.g. ear infection, upper airway infection)[19], but poor tolerability of the mask can also make its use impossible.
Other options are needed to be able to offer patients adequate analgosedation and grant good conditions for the physician to perform the necessary procedure. In addition to inhalation intranasal

administration of a drug is an attractive method as it is non-invasive as well. Intranasal administration causes minimal discomfort and is easy to use in the pediatric population. The onset of action with intranasal administeration approaches that of slow intravenous medication as first-pass metabolism is avoided with most drugs used intranasally.[20]

Intranasal dexmedetomidine has shown promising features for PSA in non-invasive procedures e.g. diagnostic imaging[21]. It has also been used successfully as premedication[22]. Dexmedetomidine is a highly selective α_2-adrenoceptor agonist and has both sedative and analgesic effects [23]. It does not cause any local irritation or pain when administered intranasally.[24,25] Intranasal dexmedetomidine has relatively short onset of action when administered intranasally, 15-25minutes[21,26]. It has no significant effects on respiratory or cardiovascular system[21,25,26]. These features make dexmedetomidine an interesting drug to study with respect to PSA in the ED.

In this trial we are comparing intranasal dexmedetomidine with inhaled N_2O to find out if intranasal dexmedetomidine could offer equally good circumstances (i.e. analgesia and sedation) for performing necessary procedures in the emergency department and if it could be an option for inhaled N_2O for PSA.

Children between 3 and 15 years of age who come to the pediatric ED at ALB with extremity fracture/luxation that require reduction or burn less than 4% of body surface can participate in this study. Previously healthy, Swedish speaking children.
Children with ASA classification ≥ III, current respiratory tract infection, ear infection, active and uncontrolled vomiting, impaired level of consciousness, uncontrolled hypotension, cerebrovascular conditions will be excluded from this study. Hypersensitivity for dexmedetomidine or nitrous oxide also excludes the patient from this trial. Other contraindications named in the product resume for the trial medicines would categorize the patient as ASA ≥3 and therefore not suitable for this trial.
Patients are randomized in to two groups: 1. intranasal dexmedetomidine, 2. Inhaled N_2O. This is an open-label study. Other than the trial medicine the patient will receive exactly same care as routine in the ALB. During the sedation period patients will be monitored with pulse oximetry and heartrate continuously.

Primary outcome is pain, maximum level of the pain during the procedure compared to pain before sedation. Pain will be assessed with FLACC (Facies, Legs, Activity, Cry, Consolability) scale[27] by a trained nurse. The secondary outcome is sedation, patient's/guardian's satisfaction and doctor's opinion about the feasibility of the procedure. Patient/guardian(s) will receive a short questionnaire to fill. Sedation level will be assessed with Ramsay sedation scale[28].

78 patients in each group (total 156 patients) will give the trial power of 80,7% to yield a statistically significant result. This computation is based on the assumption, that the mean difference is 2,0 and the common within-group standard deviation is 2,5 on the FLACC scale.
This trial will be started during spring 2017 as acceptance from Medical Product Agency and Regional Ethical Board Stockholm is received. The trial will last until the end of 2020.

References please see Supplement 1.

6.1. General information

6.1.2 Name and address of the sponsor

Malin Rydh Rinder
Funktionsområdeschef Akutsjukvård Barn, Överläkare, Med Dr
Astrid Lindgrens Barnsjukhus
Karolinska Universitetssjukhuset
Solna 17176 Stockholm
Tel +46739870384
malin.ryd-rinder@sll.se

6.1.3 Name and title of the person(s) authorized to sign the protocol

Anna Nikula, barnläkare, biträdande överläkare
anna.nikula@fimnet.fi

6.1.4 Name, title, address, and telephone number(s) of the sponsor's medical expert for the trial.

Lisa Kurland, Professor
Institutionen för medicinska vetenskaper
Örebro universitet
701 82 Örebro
tel. +4619303680
Lisa.Kurland@oru.se

Stefan Lundeberg, öl, med Dr
Smärtbehandlingsenheten
Barnanestesikliniken
Astrid Lindgrens Barnsjukhus
Karolinska Universitetssjukhuset
Solna 17176 Stockholm
Tel +46851770000
stefan.lundeberg@sll.se

Veronica Lindström, specialistsjuksköterska, Med.dr, post.doc.
institutionen NVS, sektionen för omvårdnad
Karolinska Institutet
Alfred Nobels allé 23 C4, 141 83 Huddinge
Tel + 46703428157
veronica.lindstrom@ki.se

6.1.5 Name and title of the investigator(s) who is (are) responsible for conducting the trial, and the address and telephone number(s) of the trial site(s).

Anna Nikula
Barnläkare, biträdande överläkare
Astrid Lindgrens Barnsjukhus
Karolinska Universitetssjukhuset
Solna 17176 Stockholm
Tel +46700028410

6.1.6 Name, title, address, and telephone number(s) of the qualified physician, who is responsible for all trial-site related medical (or dental) decisions (if other than investigator).

Anna Nikula
Barnläkare, biträdande överläkare
Astrid Lindgrens Barnsjukhus
Karolinska Universitetssjukhuset
Solna 17176 Stockholm
Tel +46700028410
anna.nikula@fimnet.fi

Informed Consent of Trial Subjects

Information about the study is given after doctor's examination and assessment that procedure is necessary and can be performed in the ED. Assessment will be done on medical grounds and according the guidelines.
Patient and guardian(s) are given information about the trial by a doctor (responsible for patient's care / specialist in the ED / primary investigator), for the child on the level he/she understands. Guardian(s) and children older than 7 years of age are given a written information (see Supplement 2b). They will be provided necessary time to go through the information and to ask supplementary questions. They will be informed that they at any point have the right to end their child's participation without giving any reason or explanation and without it affecting the necessary treatment.

Guardian(s) is asked to sign a consent (see Supplement 2c) if willing to participate in the trial. As it is a emergency situation both guardians may not be available. The other guardian will be contacted by telephone, information about the study and possibility for questions will be given as for the guardian present. If the guardian not present gives oral consent that will be noted on the consent form and verified with a signature from guardian who is present, as well as by the doctor giving the information about the trial. The guardian who is not present will be asked to sign the consent later. A copy of the consent form will be provided with a prepaid envelope with address.

Patient/guardian will receive a copy of the written information and the signed consent.

Consent will be signed before any procedures related to this trial are done.

Records and reports

Results of the safety and efficacy will be reported to EudraCT database at latest 6 months after the end of the trial.

Declaration of End of Trial Notification will be done within 90 days from termination of the trial. With early termination notification to Medical Product Agency if the reason for termination is trial safety.

In the case of early termination, the sponsor will notify the end of the trial to the Medical Product Agency and the Reginal Ethical Board Stockholm immediately and at the latest within 15 days after the trial is halted, clearly explain the reasons, and describe follow-up measures, if any, taken for safety reasons.

For reporting suspected adverse events please see 6.8.3.

A "serious breach" will be reported to Medical Product Agency immediately or latest within seven (7) days. The sponsor is responsible to judge the consequences of the breach and thereby decide whether Medical Product Agency will be informed.

CRF (Supplement 3) and all other essential documents will be archived for 10 years.

6.2 Background Information

6.2.1 Name and description of the investigational product(s).

Dexmedetomidine is a selective α-2 adrenergic agonist with sedative and analgesic properties. It acts by binding to G-protein coupled to α-2 adrenergic receptors, which are found in central, peripheral and autonomic nervous systems and also in various vital organs and blood vessels throughout the body. The site of action for sedative effects of dexmedetomidine is locus ceruleus and is mediated by hyperpolarization of noradrenergic neurons thus inhibiting noradrenaline release and inhibiting activity in descending medullospinal noradrenergic pathways.[29]

A bioavailability study of intranasal dexmedetomidine showed that the pharmacological effects were similar with both routes of administration, but their onset was more rapid after intravenous administration.[24] In the same study the elimination half-life was shown to be 114 (107–151) for intra nasal administration and 115 (99–145) min for intravenous administration. Bioavalability for intranasal dexmedetomidine was shown to be 63%. Pharmacokinetic data in children is limited. Half-life of dexmedetomidine in children (1 month to 17 years) appears similar to that seen in adults.

The method of administration for dexmedetomidine is intravenous according to in the SmPC. But ePed (Erfarenhet och evindensbaserad database för barnläkemedel) provide information for intranasal use of dexmedetomidine. Please see Supplement 4.

Nitrous oxide (N_2O) is an inorganic inhalation agent.[30] It is a colorless and odorless gas, which makes it acceptable to pediatric patients. It is rapidly absorbed via pulmonary vasculature directly into the bloodstream and does not combine with haemoglobin or any of the body tissues. This extremely low solubility in blood produces its rapid onset and offset of action. Elimination occurs by expiration in a manner that is precisely the reverse of uptake and distribution, and its low solubility allows nitrous oxide to be removed rapidly.[30] N_2O has sedative, anxiolytic, and amnestic properties. N_2O is used as routine for PSA in the ED at Astrid Lindgren Children's hospital. For guideline please see Supplement 5.

6.2.2 A summary of findings from nonclinical studies that potentially have clinical significance and from clinical trials that are relevant to the trial.

Initially dexmedetomidine has been used for sedation and analgesia in intensive care, but recently its use has been extended to various other clinical situations. In pediatric population it has shown to be successful as premedication and as a sedative for non-invasive procedures i.e. imaging studies.
As premedication[26] and sedative for computed tomography[21] it has been administered intranasally. Intranasal dexmedetomidine has relatively short onset of action when administered intranasally, 15-25 minutes[26] and that makes it a convenient drug for PSA.

Several studies with intravenous[31,32,33,34] and intranasal[21,22,35,36] dexmedetomidine for procedural sedation and premedication have shown the safety of dexmedetomidine. These studies show that dexmedetomidine has minimal effect on cardiorespiratory function. No clinically significant changes on systolic blood pressure, heart rate, respiratory rate or saturation was reported. Clinically significant changes were mostly defined as change of 20% or 2SD below or above normal limits for age or saturation below 95%.

In a study with 62 children (age 2-6 years) comparing intranasal and buccal administration of dexmedetomidine (1µg/kg dose) no patient had respiratory depression, bradycardia, or desaturation.[35] Another study with 60 patients (children with mean age 17,5 ± 9,5 months (mean weight 10,7 ± 2,8 kg)) intranasal administration of dexmedetomidine (total dose 3,5 µg/kg) showed one case of hypoxia and hemodynamic variability in 17% classified as minor risk.[21] Yuen VM et al in their study compared 1 and 2 µg/kg intranasal dose of dexmedetomidine showed no effect on oxygen saturation levels.[37]

6.2.3 Summary of the known and potential risks and benefits, if any, to human subjects.

For undesirable effects of dexmedetomidine and N_2O please see respective product resume (Supplement 6 and 7). Dexmedetomidine is used in smaller dose for sedation than for anesthesia risk for adverse events is smaller. And even though the medication is not administered the way described in the product resume there is good evidence of the safety and effectivity of intranasal administration of dexmedetomidine.[21,22,35,36] For more information please see 6.2.2 and 6.2.4

In the product resume for dexmedetomidine common side-effects with IV infusion are hypotension, bradycardia, respiratory depression, nausea, dry mouth, hyper-/hypoglycaemia, agitation. With intranasal administration no significant bradycardia or hypotension has been shown. Nor any other of the above mentioned side-effects has been reported with intranasal administration. (please see 6.2.2).

Most common side-effect with N_2O is nausea and vomiting. A large French survey reported 4,4% adverse events, of which nausea/vomiting and agitation/euphoria accounted for 87%.[16] In the same prospective survey 0,03% serious adverse events were directly related to N_2O (consciousness disorder, vomiting, bradycardia, vertigo, headache, nightmares, sweating, somnolence) were reported, other studies have shown the incidence for serious adverse events 0 - 0,33%[15]. N_2O has little effect on respiratory system. Studies with 50% N_2O only (no concomitantly administration of sedative/opioid) show no desaturation or apnea. Onody et al reported two cases of serious adverse events (cardiac arrest and O_2 desaturation) with inappropriate use of the administration device and insufficient surveillance.[16] Diffuse hypoxia can be seen when N_2O is discontinued, the gradient favours the movement of N_2O from the blood into the alveolus and diluting out the oxygen. To prevent that supplemental oxygen will be provided after discontinuation of N_2O for 3-5 minutes. There are few reports of seizures[38] during N_2O and even one case report of laryngospasm[39]. All these cases have been children under the age limit of our study and the N_2O concentration has been higher than 50%.

In addition to the possible side effects of the medications explained above can of course pain, distress and discomfort be caused by the procedure itself. But the procedures are medically necessary and need to be carried through weather patient is included in the study or not. As is routine today at Astrid Lindgren Children's hospital ED the procedure will be disrupted if effective sedation or analgesia is not reached. In which case, other methods for sedation and analgesia will be used (i.e. anesthesiologist can provide deeper sedation and analgesia either in the ED or in the operation theater).

Both medications have minimal circulatory and respiratory effect. The patients are monitored for oxygen saturation and heart rate to be able to detect possible circulatory and respiratory issues. If any aforementioned issues would occur the trained staff in the ED will provide first hand treatment with supplemental oxygen and ventilation if needed and circulatory support. N_2O administration will be discontinued if that is the drug used. According to routine anesthesia team will be alarmed. All the equipment for airway and circulatory management will be on hand in the procedure room.

Benefits versus risks of the study

The expected value of this study is to improve knowledge regarding efficacy of inhaled N_2O and dexmedetomidine when used for sedation and analgesia during procedures for children. It is well known that the effect of different drugs used in this field are less studied regarding use in children. Nevertheless, it is even more important that children can be treated safely with drugs during painful situations.

The route of treatment inhalation and intranasal application is of special interest for use in the pediatric population since it does not require venous access which is painful itself.

There are studies showing a better safety profile for using dexmedetomidine intranasally. The incidence of effects on blood pressure and circulation is lower than for intravenous use why the setup of this study is likely to have less risks than using intravenous injections of sedative and analgesic drugs.

Since these both drugs already are in use in the study hospital in a controlled manner (see Supplement 5 and 8) the risk of handling and administration and evaluation is judged to be low and the value of expanding the knowledge of the efficacy of the treatment for painful procedures is stronger than the risk for severe side effects.

The alternative method for sedation and analgesia if inhaled N_2O and intranasal dexmedetomidine are not potent enough for the procedure is more profound anaesthesia which has potentially more risks and requires more resources.

In conclusion, the benefit-risk assessment of both drugs used in this study is considered to be positive in the population in question. Both drugs used in this study are considered to give pain alleviation and sedation. And both drugs have been shown to have a good safety profile in several studies as well as in routine clinical use in the study hospital.

6.2.4 Description of and justification for the route of administration, dosage, dosage regimen, and treatment period(s).

Intranasal administration is easy and convenient way of administrating a medicine.[40] It does not require intravenous access; a needle stick is often frightening for a child. Dexmedetomidine is odorless and tasteless, and can be administered without discomfort. It is easy to use in the pediatric population. The highly vascularized nasal mucosa and the olfactory tissue in direct contact with the central nervous system allow nasally administered drugs to be rapidly transported into the bloodstream and brain, with onsets of action approaching that of intravenous therapy. First-pass metabolism via the liver is also avoided and that results in high bioavailability of many medications.[41]

An atomizer has been shown to deliver a consistent volume of the drug and it has shown to increase patient acceptance compared to nasal drops. But the administration method did not affect the effectivity of the drug.[42] We have chosen to use an atomizer to administer dexmedetomidine intranasally because it is a well-accepted method and also recommended in ePeds database instructions (see Supplement 4).

Dose 2,0 mikrograms/kg will be used and this is chosen after current practice for premedication in Astrid Lindgren Children's hospital (see Supplement 8) and this dose for procedures is recommended in ePed database (see Supplement 4) This This dose has also been shown to produce safe and efficient sedation.[37]

Dexdor® 100 µg/ml concentration is used without further dilution. A 1 ml syringe with a special soft plug for nasal administration (MAD Nasal™) will be used. The volume should not exceed 0,2 ml per nostril. In this patient group 10 – 15 kg it could mean maximum one spraying per nostril.

N_2O is an inhalation gas and therefore will be inhaled through a mask. Concentration 50% N_2O : 50 % O_2 has been chosen as it is the normal routine at Astrid Lindgren Children's hospital (see Supplement 5) and also because it has been shown to be effective and safe[16]

There will be only one treatment occasion. The planned procedure will be done during the emergency department visit. Patient will receive one dose of dexmedetomidine or inhale 50% N_2O during the procedure, inhalation will be started 3-5minutes prior and ended as the procedure is over.

6.2.5 A statement that the trial will be conducted in compliance with the protocol, GCP and the applicable regulatory requirement(s).

This study will be conducted according to protocol and current regulations LVFS 2011:19, ICH GCP and the latest version of Helsinki declaration.

6.2.6 Description of the population to be studied.

Children at the age of 3-15 years who present to the emergency department with extremity fracture/luxation that require reduction or burn less than 4% of body surface area. Previously healthy Swedish speaking patients.

6.2.7 References to literature and data that are relevant to the trial, and that provide background for the trial.

Please see Supplement 1

6.3 Trial Objectives and Purpose

The overall aim is to identify other possibilities for non-invasive, safe and efficient procedural analgesia and sedation to be used for children in the emergency department.

The objective of this study is to evaluate whether intranasal dexmedetomidine is equally good as nitrous oxide (N_2O) among children between 3 and 15 years of age with minor injuries with respect to analgesia during procedure measured by FLACC in a prospective randomized open-label study.

We are hoping that the results from this study can be used for new recommendations about using dexmedetomidine for PSA for painful procedures in combination with local anesthetics in the patient group mentioned above.

6.4 Trial Design

6.4.1 A specific statement of the primary endpoints and the secondary endpoints, if any, to be measured during the trial.

Primary outcome is pain measurement during the procedure, pain during the procedure compared to the pain right before procedure start. Pain will be assessed with FLACC[27] (Face, Legs, Activity, Cry, Consolability scale) and change of 1 point on the scale of 0-10 is considered as significant change. For closer explanation of the behavioural components please see Supplement 9.

FLACC was initially validated by Merkel and colleagues with children age 2mo – 7 yr postoperatively.[27] Since then it has been tested and validated in several settings (e.g. postoperatively, PICU, trauma unit and oncology unit)[43] A translation to Swedish and validation of the Swedish version was done by Nilsson et al.[44] This study also concluded that FLACC can reliably be used for assessing procedural pain, though the study was carried through with children age 5-16 years. There are several studies on procedural sedation that have used FLACC score as pain assessment scale. As FLACC was initially validated for children from 2 months of age and its reliability in procedural sedation also is shown we have chosen this pain scale for the assessment tool in our study.

Secondary outcome is sedation score during the procedure with the baseline defined as score before administration of medicine. Other secondary outcomes are patient's/guardian's satisfaction and doctor's opinion about the feasibility of the procedure. Patient/guardian(s) will receive a questionnaire with few questions. Doctor's opinion will be recorded on the CRF.

To assess the sedation Ramsay sedation score[28] will be used, a change of 1 point on the scale of 1-6 is considered as significant change. For closer explanation of the components of this scale please see Supplement 10. Ramsay scale is translated to Swedish by SFAI (Svensk förening för anestesi och intensivvård).

The Ramsay sedation scale was first published 1974[28] and today it is one of the most widely used tools for observationally based sedation assessment. Ramsay sedation score is not validated for children, but it is widely used sedation score for children and has been used in many studies with intranasal dexmedetomidine[21,37,45].

6.4.2 A description of the type/design of trial to be conducted (e.g. double-blind, placebo-controlled, parallel design) and a schematic diagram of trial design, procedures and stages.

A prospective, randomized, open-labeled clinical trial.
Patients will be randomized into two groups; group 1: intranasal dexmedetomidine, group 2: inhaled N_2O. 156 patients will be included in the trial, 78 in each group.

1. Paracetamol
    - All patients will receive oral paracetamol 40 mg/kg (max 2 g) on the arrival to the ED or at least 1-1,5 hours before procedure. This is a routine treatment of pain for all patients with injuries in the ED at ALB.
2. Doctor's examination and assessment that procedure is necessary and can be performed in the ED. Assessment will be done on medical grounds and according the guidelines.

3. Patient meets the inclusion and exclusion criteria
    - Inclusion criteria
        - 3-15 years of age
        - extremity fracture/luxation that require reduction or burn less than 4% of body surface area
        - Previously healthy
        - Swedish speaking
    - Exclusion criteria
        - ASA classification ≥ III (see Supplement 11)
        - Current respiratory tract infection
        - Ear infection
        - Sinusitis
        - Pertussis within 6 months
        - Any symptoms of breathing difficulty
        - Active and uncontrolled vomiting
        - Impaired level of consciousness
        - Psychiatric issues
        - Hypersensitivity for dexmedetomidine or N_2O.
        - Further contraindications named in the product resume for the trial medicines would categorize the patient as ASA ≥III and therefore not suitable for this trial.
            - Advanced heart block (grade 2 or 3) unless paced
                - ECG will be done to exclude AV block II/III
            - Known and untreated uncontrolled hypotension
                - Bloodpressure is not to be measured on patients with no history
            - acute cerebrovascular conditions (=patient with any acute neurological symptoms)

4. Information about the trial
    - Information will be provided by treating physician / specialist Dr in the ED / primary investigator
    - Spoken and written information will be provided
        - For written information please see Supplement 2b

5. Written consent
    - Please see page 8 in the protocol
    - Please see Supplement 2b

6. Randomization
    - The patients will be randomized in a double fashion to minimize bias.
    - Randomization will be done by a person not participating the trial. Name of medicine will be written on a paper and put in an envelope which will be sealed and numbered. Patient will choose the envelop from those that are left.

7. ECG
    - Assessed by the investigator in charge to exclude AV block II/III.

8. Administration of trial medicine
   GROUP 1: intranasal dexmedetomidine (Dexdor® 100microg/ml) 2,0 microg/kg
   GROUP 2: inhaled N_2O (50% N_2O : 50% O_2)

- Mask will be held by a nurse during the whole procedure
- N_2O will be titrated to the concentration 50% in 2-3minutes
- With fracture/luxation a break for about 3 minutes will be taken while waiting the local anesthetic to have an effect
- After the discontinuation of N_2O supplemental O_2 will be given for 2-3 minutes

9. Monitoring and observation
   - continuous SpO_2 and heartrate
     - beginning from the administration of trial medicine
     - until sedation score 1 according to Ramsay is reached again
   - Pain (FLACC) and Sedation (Ramsay) assessment
     - at 0 – 5 – 10 min from administration of study medicine and continue every 5 minutes until Ramsay score 2 is reached
     - at the start of the procedure and every 5min under the procedure
     - after procedure every 10 minutes until the patient has recovered and reached Ramsay score 1

10. Procedures will be will be carried out according the normal routines

Burn 1. Gauze soaked with buffered lidocaine (10ml Xylocain® 10mg/ml + 2ml $NaHCO_3$) will be put on top of the burn area (maximum lidocaine dose 5mg/kg)
- IN medication: at the same time/right after administration
- N_20: 20-30 minutes before

2. Cleaning and dressing of the burn as per routine protocol
- as sedation score 2 on Ramsay sedation scale is reached or 30 minutes from administration of intranasal medicine

Fracture 1. Local anesthesia with buffered lidocaine (10ml Xylocain® 10mg/ml + 2ml $NaHCO_3$) is infiltrated on the fracture site with needle and syringe

maximum dose lidocaine without adrenalin 5mg/kg

maximum dose lidocaine with adrenalin 7mg/kg
- as sedation score 2 on Ramsay sedation scale is reached or 30 minutes from administration of intranasal medicine

2. Reduction of the fracture / luxation as per routine protocol
- 5 minutes after local anesthesia application

11. Patient will be able to leave the emergency department when Ramsay score 1 is reached and he/she has returned to his/hers habitual condition.

And as the management of the injury is completed according the normal routine and further information and possible follow-ups are given.

6.4.3 A description of the measures taken to minimize/avoid bias, including:

(a) Randomization.

The patients will be randomized in a double fashion to minimize bias.
Randomization will be done by a person not participating the trial.
Randomization will be done in blocks of 10 subjects (5 from both arms), except one block with 12 subjects. A list for randomization will be created after a random draw up. Envelopes will be filled with information according to randomization list and numbered. Envelopes will then be used in number order.

6.4.4 A description of the trial treatment(s) and the dosage and dosage regimen of the investigational product(s). Also include a description of the dosage form, packaging, and labelling of the investigational product(s).

Paracetamol 40 mg/kg orally (max 2 g). Dose 40 mg/kg is used for pain management as loading dose according to clinical routine at Astrid Lindgren Children's hospital (see Supplement 12 page 30) which are based on the Swedish national guidelines[46].

Buffered lidocaine (10ml Xylocain® 10mg/ml + 2ml $NaHCO_3$) is used for local anesthesia. Maximum dose of lidocaine without adrenalin is 5 mg/kg and with adrenalin is 7 mg/kg (see Supplement 12 page 29)

Intranasal dexmedetomidine 2,0 micrograms/kg as one dose.
Dexdor® 100 μg/ml concentration is used without further dilution. A 1 ml syringe with a special soft plug for nasal administration (MAD Nasal™) will be used.

Inhaled N_2O is a gas provided by AGA to Astrid Lindgren Children's hospital. N_2O will be titrated to the concentration of 50% N_2O : 50% O_2 by a trained nurse.
A contract with the hospital pharmacy will be made for labelling and packaging the investigational products. The commercial product of dexmedetomidine (Dexdor® 100mikrog/ml) will be used. N_2O will be delivered by the producer (AGA) as normally for the use in the ED.
The labelling will include study number, responsible investigator and statement "for clinical testing only". As well information about the drug: name, batch number, expiry date.

6.4.5 The expected duration of subject participation, and a description of the sequence and duration of all trial periods, including follow-up, if any.
The whole process from administration of the drug and finish of the procedure will take about 30-60 minutes. In addition to that a recovery time up to one hour can be expected.
No further follow-ups are necessary, except the follow-up required for the injury and that will follow the normal guidelines.

6.4.6 A description of the "stopping rules" or "discontinuation criteria" for individual subjects, parts of trial and entire trial.

The procedure will be stopped if it cannot be carried through because of insufficient pain relief judged by doctor, patient or guardian. As well as if any adverse events (e.g. breathing problems, desaturation, vomiting) occur.
Furthermore, if patient/guardian decides not to continue the procedure.
Eventually if administration of the intranasal trial medicine must be stopped because of local irritation, but that will only be the case when the dose needs to be divided into both nostrils as nasal administration only takes 1-2 seconds. Or if inhalation of N_2O needs to be stopped because the subject is unwilling to inhale/use the mask.

6.4.7 Accountability procedures for the investigational product(s), including the placebo(s) and comparator(s), if any.

Marketed drugs will be used. Placebo cannot be used since the procedure is painful and cannot be carried out without an adequate treatment.
Both drugs are used in clinical practise (please see Supplements 4,5,8).
N_2O is provided to hospital by AGA by routine and this gas will be used in this trial as in normal situations.
Dexmedetomidine (Dexdor® 100µg/ml) will be ordered from the hospital pharmacy with the labelling presented in section 6.4.4.

6.4.8 Maintenance of trial treatment randomization codes and procedures for breaking codes.

Envelopes for randomization codes will be kept safely (locked in) in the ED.

6.4.9 The identification of any data to be recorded directly on the CRFs (i.e. no prior written or electronic record of data), and to be considered to be source data.

Please see the CRF attached (Supplement 3)

6.5 Selection and Withdrawal of Subjects

6.5.1 Subject inclusion criteria.

- 3-15 years of age
- extremity fracture/luxation that require reduction or burn less than 4% of body surface area
- Previously healthy (ASA I and II)
- Swedish speaking

6.5.2 Subject exclusion criteria.

- ASA classification ≥ III (see Supplement 11)
- Current respiratory tract infection
- Ear infection
- Sinusitis
- Pertussis within 6 months
- Any symptoms of breathing difficulty
- patients with ongoing vomiting during the visit at the ED
- Impaired level of consciousness
- patients with diagnosed with psychiatric disorder
- Hypersensitivity for dexmedetomidine or N_2O.
- Further contraindications named in the product resume for the trial medicines would categorize the patient as ASA ≥III and therefore not suitable for this trial.
    - Advanced heart block (grade 2 or 3) unless paced
        - ECG to exclude AV block II/III
    - Known and untreated uncontrolled hypotension
        - Bloodpressure is not to be measured on patients with no history
    - acute cerebrovascular conditions (=patient with any acute neurological symptoms)

6.5.3 Subject withdrawal criteria (i.e. terminating investigational product treatment/trial treatment) and procedures specifying:

(a) When and how to withdraw subjects from the trial/ investigational product treatment.

Patients will not be included in the study if they don't meet the inclusion criteria or meet any of the exclusion criteria. The study includes only one treatment occasion with the investigational drug. Patient/guardian can at any time choose to stop participation in this trial and will then be treated according the normal routines of the hospital.

(b) The type and timing of the data to be collected for withdrawn subjects.

CRF will be used for collecting data and data will be registered until subject withdraws. The reason for withdrawal will be registered if patient is willing to provide one.

(c) Whether and how subjects are to be replaced.
Withdrawn subjects will be replaced so that planned number of subjects (156=78/group) is reached

(d) The follow-up for subjects withdrawn from investigational product treatment/trial treatment.

Patient will be treated as per normal routines if trial needs to be discontinued.
If any adverse events occur patient will be treated and followed up according to the need and type of side effect. All adverse events will be recorded and reported to Medical Product Agency.

6.6 Treatment of Subjects

6.6.1 The treatment(s) to be administered, including the name(s) of all the product(s), the dose(s), the dosing schedule(s), the route/mode(s) of administration, and the treatment period(s), including the follow-up period(s) for subjects for each investigational product treatment/trial treatment group/arm of the trial.

Paracetamol
40 mg/kg orally (oral suspension) (maximum dose 2 g) on arrival to the Emergency Department or at least 1-1,5 hours before procedure.
Buffered **lidocaine** (10ml Xylocain® 10mg/ml + 2ml $NaHCO_3$) is used for local anesthesia. Maximum dose of lidocaine without adrenalin is 5 mg/kg and with adrenalin is 7 mg/kg
Buffered lidocaine will be injected locally in the fracture/luxation area when Ramsay score 2 is reached and 5minutes prior procedure. With burns a compress soaked in buffered lidocaine will be put on the burn 20-30 min before the procedure.

Dexmedetomidine
2,0 mikrograms/kg intranasally, one dose.
Dexdor® 100 µg/ml concentration is used without further dilution. A 1 ml syringe with a special soft plug for nasal administration (MAD Nasal™) will be used.
Monitoring with SpO_2 and pulse until Ramsay scale 1 after the procedure is reached and child has returned to his/her normal behaviour.

N_2O will be administered through a mask that is held by a trained nurse. N_2O will be inhaled through the whole procedure, with an exception the time waiting for local anesthesia to have effect when doing fracture/luxation reduction. N_2O will be titrated to the concentration 50% N_2O : 50% O_2 in 2-3 minutes. After the discontinuation of N_2O supplemental O_2 will be provided for 2-3 minutes. This will follow the local guideline at Astrid Lindgren Children's hospital for N_2O sedation (please see Supplement 5). Monitoring with SpO_2 and pulse until Ramsay scale 1 after the procedure is reached and child has returned to his/her normal behaviour.

6.6.2 Medication(s)/treatment(s) permitted (including rescue medication) and not permitted before and/or during the trial.

2 hours must have passed from administration of other sedatives or opioids.
No rescue medicine will be used. If effective analgesia and sedation is not reached the procedure will be stopped and other methods to perform the necessary treatment will be used (please see 6.2.3)

6.6.3 Procedures for monitoring subject compliance.

Not needed in this trial.

6.7 Assessment of Efficacy

6.7.1 Specification of the efficacy parameters.

FLACC and Ramsay scales

To assess the effectivity on pain management during the procedure FLACC (Face, Legs, Activity, Cry, Consolability scale) scale will be used.
The sedation effectivity of the drugs used will be assessed with Ramsay sedation scale.

For further information on FLACC and Ramsay sedation scale please see point 6.4.1.

6.7.2 Methods and timing for assessing, recording, and analysing of efficacy parameters.

Timing for assessment please see 6.4.2.
Data will be recorded on CRF, please see Supplement 3.

6.8 Assessment of Safety

6.8.1 Specification of safety parameters.

Adverse event (defined in Article 2(m) of Directive 2001/20/EC)
Any untoward medical occurrence in a patient or clinical trial subject administered a medicinal product and which does not necessarily have a causal relationship with this treatment.

Adverse reaction (defined in Article 2(n) of Directive 2001/20/EC)
All untoward and unintended responses to an investigational medicinal product related to any dose administered.

Serious adverse event or serious adverse reaction (defined in Article 2(n) of Directive 2001/20/EC)
Any untoward medical occurrence or effect that at any dose results in death, is
life-threatening, requires hospitalization or prolongation of existing hospitalization, results in persistent or significant disability or incapacity, or is a congenital anomaly or birth defect;

Unexpected adverse reaction (defined in Article 2(p) of Directive 2001/20/EC)
An adverse reaction, the nature or severity of which is not consistent with the applicable product information (e.g. investigator's brochure for an unauthorized investigational product or summary of product characteristics for an authorized product)

Suspected unexpected serious adverse reaction (SUSAR)
An untoward and unintended response to a study drug, which is not listed is the applicable product information, and meets one of the following serious criteria: results in death, is life-threatening, requires hospitalization or prolongation of an existing hospitalization, results in persistent or significant disability or incapacity, or is a congenital anomaly or birth defect

Adverse events and reactions will be classified as serious if it results in death, is life-threatening, requires hospitalization or prolongation of existing hospitalization, results in persistent or significant disability or incapacity.

The Reference Safety Information and known side effects are contained in the Summary of product characteristics (in Swedish, if needed an English translation can be provided)

Biverkningar: dexmedetomidin infusion
Biverkningarna är rangordnade efter frekvens enligt följande: Mycket vanliga (≥ 1/10), vanliga (≥1/100,<1/10), mindre vanliga (≥1/1 000, <1/100), sällsynta (≥1/10 000, <1/1 000), mycket sällsynta (<1/10 000).
Metabolism och nutrition
Vanliga: Hyperglykemi, hypoglykemi
Mindre vanliga: Metabolisk acidos, hypoalbuminemi
Psykiska störningar
Vanliga: Agitation
Mindre vanliga: Hallucination
Hjärtat
Mycket vanliga: Bradykardi*
Vanliga: Myokard ischemi eller hjärtinfarkt, takykardi
Mindre vanliga: AV-Block I, minskad hjärtminutvolym

Blodkärl:
Mycket vanliga: Hypotension*, hypertoni*
Andningsvägar, bröstkorg och mediastinum
Vanliga: Andningsdepression
Mindre vanliga: Dyspné, apné
Magtarmkanalen
Vanliga: Illamående, kräkningar, muntorrhet
Mindre vanliga: Svullen buk
Allmänna symtom och/eller symtom vid administreringsstället
Vanliga: Abstinenssyndrom, hypertermi
Mindre vanliga: Läkemedlet ineffektivt, törst

Biverkningar vid dikväveoxid inhalation
Biverkningarna är rangordnade efter frekvens enligt följande: Mycket vanliga (≥ 1/10), vanliga (≥1/100,<1/10), mindre vanliga (≥1/1 000, <1/100), sällsynta (≥1/10 000, <1/1 000), mycket sällsynta (<1/10 000).
Centrala och perifera nervsystemet
Mycket sällsynta: Myelopati, polyneuropati
Öron och balansorgan
Mindre vanliga: Tryckkänsla i mellanörat
Magtarmkanalen
Vanliga: illamående, kräkningar
Mindre vanliga: Uppblåsthet, ökad gasvolym i tarmarna
Allmänna symptom och/eller symtom vid administreringsstället
Vanliga: Yrsel, berusningskänsla*

6.8.2 The methods and timing for assessing, recording, and analyzing safety parameters.

Saturation and heart rate will be continuously monitored with pulse oximetry.
This monitoring will be carried out during the whole sedation time, i.e. from the administration of intranasal drug or beginning of the N_2O treatment until the patient has returned to Ramsay scale 1.

All adverse events and reactions will be recorded on CRF and in the patient records. Following information about the event will be recorded: symptoms will be described (observation and reported by patient), severity and the possible causal relationship between the event and the IMP.

The severity of the incident will be assessed to be minor, moderate or major. The incident will also be defined as serious or non-serious (see above). These assessments will be done by the investigators of this trial. The possible relationship between the event and the IMP will be assessed (not-related, possibly related, likely related) by the investigator.

6.8.3 Procedures for eliciting reports of and for recording and reporting adverse event and intercurrent illnesses.

All adverse events whether they have causal relationship to IMPs or not will be reported.

The investigator in charge of this trial (Anna Nikula) will report all suspected serious adverse events to the sponsor (Malin RydhRinder) within 24 hours the event has come to her knowledge. The connection with investigational product will be assessed.
All suspected unexpected serious adverse events that are deadly or life-threatening will be reported by the sponsor (Malin RydhRinder) to Medical Products Agency and Ethical Review Board Stockholm within seven (7) days and complementary information within eight (8) days after the preliminary report. All other suspected unexpected serious adverse events will be reported within fifteen (15) days.
In addition to above mentioned a yearly report about adverse events will be filed to Medical Products Agency and Ethical Review board Stockholm as long as the clinical trial is active.

A "serious breach" is a violation or deviation of the protocol which is likely to effect to a significant degree the safety or integrity of the subjects of the trial; or the scientific value of the trial. A "serious breach" will be reported to Medical Product Agency immediately or latest within seven (7) days. The sponsor is responsible to judge the consequences of the breach and thereby decide whether Medical Product Agency will be informed.

6.8.4 The type and duration of the follow-up of subjects after adverse events.

Patients with suspected adverse effects will be monitored at the emergency department. The duration of the observation will depend on the type and duration of the adverse event and if necessary patient will be admitted for further monitoring.

6.9 Statistics

6.9.1 A description of the statistical methods to be employed, including timing of any planned interim analysis(ses).

Comparison between treatment groups will be performed using t-test, no interim analysis is planned. The study is intention to treat. Comparisons will be performed on group level between those eligible and those included in the trial.

6.9.2 The number of subjects planned to be enrolled. In multicentre trials, the numbers of enrolled subjects projected for each trial site should be specified. Reason for choice of sample size, including reflections on (or calculations of) the power of the trial and clinical justification.

This study will have power of 80% to show that the mean for dexmedetomidine is at least as high as the mean for nitrous oxide. This assumes that the means for the nitrous oxide and dexmedetomidine populations are are precisely equal (at 6.00), with a common within-group standard deviation of 2.50, that a difference of 1.00 points or less is clinically unimportant, that the sample size in the two groups will be 78 and 78, and that alpha (1 tailed) is set at ,05.
Formally, the null hypothesis is that the mean for dexmedetomidine is 1.00 points lower than the mean for N_2O, and the study has power of 80,0% to reject this null. Equivalently, the likelihood is

80,0% that the 95,0% confidence interval for the mean difference will exclude a difference of 1.00 points in favor of nitrous oxide.

6.9.3 The level of significance to be used.

Please see 6.9.2

6.9.4 Criteria for the termination of the trial.

The trial will be completed at the last visit of the last subject.
If several serious adverse occur, patients are difficult to enrol or the time to reach the planned number of subjects is prolonged the trial will be terminated earlier than planned.
As the investigational medicine is administered only once there is no need for further treatment or follow-up.

In the case of early termination, the sponsor will notify the end of the trial to the Medical Product Agency and the Reginal Ethical Board Stockholm immediately and at the latest within 15 days after the trial is halted, clearly explain the reasons, and describe follow-up measures, if any, taken for safety reasons.

6.9.5 Procedure for accounting for missing, unused, and spurious data.

Missing data will be excluded from statistical calculations on group level. Patients with missing data will be discussed separately in the results section.

6.9.6 Procedures for reporting any deviation(s) from the original statistical plan (any deviation(s) from the original statistical plan should be described and justified in protocol and/or in the final report, as appropriate).

Any non-substantial deviations from original protocol will be recorded.
Substantial deviations will be reported according the following:
According to Communication from the European Comission CT-1 2010/C82/01, 3.3. amendments to the trial are regarded as 'substantial' where they are likely to have a significant impact on: the safety or physical or mental integrity of the clinical trial participants, or the scientific value of the trial. For a more specific description of substantial amendments please see European Comission Guidance CT-1 2010/C82/0,1 3.4.1 / 119.
The sponsor will assess whether an amendment is to be regarded as 'substantial'.
Substantial amendments will be reported to Medical Product Agency and/or to Regional Ethical Board Stockholm depending on the amendment. Decision on which authority (or both) will be notified will be assessed and decided by the instructions given in CT-1 2010/C82/01, 3.5 and LVFS2011:19,7.

If patient safety is endangered the trial will discontinued and reported to the Medical Procut Agency authorities.

Deviation, if any, will be stated in the final report.

6.9.7 The selection of subjects to be included in the analyses (e.g. all randomized subjects, all dosed subjects, all eligible subjects, evaluable subjects).

Patients that meet inclusion criteria and are willing to participate in the study are randomized. All randomized patients who have received investigational medicine or the comparator and gone through the procedure will be included in the analysis.

6.10 Direct Access to Source Data/Documents
The sponsor should ensure that it is specified in the protocol or other written agreement that the investigator(s)/institution(s) will permit trial-related monitoring, audits, IRB/IEC review, and regulatory inspection(s), providing direct access to source data/documents.

The sponsor ensures that the hospital will provide direct access to all study data in case of monitoring or audit by any regulatory authority.

6.11 Quality Control and Quality Assurance

Monitoring will be carried out by a trained member from Karolinska Trial Alliance in accordance with Good Clinical Practice. This means that an independent qualified monitor will control inclusion of patients, signed consent, source data (CRF), patient safety and general execution of the trial according to the protocol. On-site monitoring will take place before, during and after the trial.

Patient's participation in a clinical trial will be recorded in the medical record. Name of the trial and patient's trial code will be registered.

Nurses and doctors involved in care of the patients participating in the trial will be informed about the trial and trained according to the need.

6.12 Ethics Description of ethical considerations relating to the trial.

Application for ethical review will be sent to Ethical Review Board Stockholm.

6.13 Data Handling and Record Keeping

All data concerning the trial will be recorded on a paper CRF (see Supplement 3) and the data that routinely is recorded to patient's medical records will be recorded also there.
Doctors who take part in patients care have access to the medical records according normal routine and Personal Data Act (Personuppgiftslag PUL1998:204).
CRF will be labelled with codes. List for decoding with patient information will be constructed and stored separately from the CRFs until the code is broken.
Filled CRF and signed consent forms will be stored in a locked closet in the department.

All data will be handled without personal data and only investigators who are part of this study will have access to data. An extern monitor will have access to the data.

All data will be handled according to Personal Data Act (Personuppgiftslag PUL1998:204)

A qualified person with GCP certification will monitor the performance of the study at repeated occasions.

Any changes on CRF will be done by primary inverstigator (Anna Nikula) and sponsor (Malin RydhRinder). All essential changes will be reported to Medical Product Agency.

An independent copy of the CRF will be held by the investigator after trial is concluded.

6.14 Financing and Insurance

The study is not sponsored by any pharmaceutical company.
All patients are covered by Pharmaceutical insurance (Läkemedelsförsäkringen).

6.15 Publication Policy

We intend to publish the result of this study in scientific journal and present in national/international congress.

6.16 Supplements

Supplement 1	References
Supplement 2a	Patient information and consent version1
Supplement 2b	Patient information and consent version2
Supplement 3	CRF
Supplement 4	Barnläkemedelsinstruktion: Dexmedetomidin nasalt 100 mikrog/mL
Supplement 5	Lustgas vid procedursmärta
Supplement 6	Product resume - dexmedetomidine
Supplement 7	Product resume – N_2O
Supplement 8	Sedering vid MR-undersökning av barn med dexmedetomidine (BANE)
Supplement 9	FLACC skala
Supplement 10	Ramsay sederingsskala
Supplement 11	ASA Physical Status Classification System
Supplement 12	Riktlinjer för smärtbehanlind vid Astrid Lindgrens Barnjukhus
Supplement 13	Changes to previous application

Stockholm 2017-06-21

Malin Rydh-Rinder
Funktionsområdeschef Akutsjukvård Barn, Överläkare, Med Dr
Astrid Lindgrens Barnsjukhus
Karolinska Universitetssjukhuset
Anna Steckséns gata 35
17 176 Solna
Tel 0739870384

Anna Nikula
Barnläkare, Biträdande överläkare
Akutsjukvård Barn
Astrid Lindgrens Barnsjukhus
Karolinska Universitetssjukhuset
Anna Steckséns gata 35
17 176 Solna
Tel 0700028410
